# When digital tracking helps or hinders: unpacking the heterogeneous effects of activity trackers on adolescent athletes through the lens of motivation and acceptance

**DOI:** 10.3389/fpsyg.2026.1813784

**Published:** 2026-04-28

**Authors:** Jixing Wu, Caizhu Gao, Hang Yin, Dayuan Piao

**Affiliations:** 1 School of Physical Education, Huaqiao University, Quanzhou, China; 2 School of Sports Science, Anshan Normal University, Anshan, China; 3 School of Physical Education, Changchun Normal University, Changchun, China

**Keywords:** adolescent athletes, digital physical activity trackers, heterogeneous treatment effects, quantile regression, self-determination theory, sports psychology, technology acceptance model, youth football

## Abstract

**Background:**

Digital activity trackers have become popular in youth training, but the evidence of their effectiveness is mixed. Most of the existing studies rely on the average effect model, ignoring the individual differences in the effect of equipment use and the psychological mechanism behind it.

**Objective:**

The purpose of this study is to explore the heterogeneous dose–response relationship between the objective use of trackers and the improvement of performance in young football players, and to analyze the mediating role of training load and the moderating role of psychological acceptance and initial physical fitness.

**Methods:**

The study followed 142 young male football players from 12 youth academies for 12 weeks. We integrated multiple sources of data, including objective usage data recorded by the device (cumulative duration/frequency), performance improvement data (ΔYo-Yo interval recovery test scores), training load data (based on RPE scores), and psychological acceptance data (based on the TAM questionnaire). The quantile regression model was used to analyze the role of each factor under different levels of performance improvement (*τ* = 0.10 to 0.90), followed by robustness tests and quantile-specific mediation and moderation analyses.

**Results:**

There was significant heterogeneity in the association: the association between cumulative duration of use and performance improvement was strongest at the low quantile (*τ* = 0.25: *B* = 2.41, *p* < 0.001) and not significant at the high quantile (*τ* = 0.90: *B* = −0.21, *p* = 0.68). Training load was only mediating the association in the middle and lower percentiles (*τ* = 0.25 indirect effect = 0.89, 95% CI [0.32, 1.65]). Psychological acceptance positively moderated the association at low performance levels, while initial physical fitness negatively moderated it at low and middle levels, which together explained the observed heterogeneity. The observational design precludes causal inferences.

**Conclusion:**

The association between tracker use and performance varied significantly among individuals. The potential benefits were most significant for athletes with lower initial fitness levels and higher psychological acceptance, achieved in part through increased training loads. Practice requires personalized application. Due to the observational study design, no causal relationship can be inferred from these findings.

## 1 Introduction

The pattern of youth sports training is undergoing profound changes, which is driven by the full integration of digital technology. Wearable digital motion trackers that provide real-time, objective biofeedback have been rapidly adopted in competitive sports systems (Ransom et al., 2025). The value of these devices lies in improving the scientific and personalized level of training, with the ultimate goal of optimizing sports performance (Passos et al., 2021). From a psychological point of view, digital sport trackers are believed to play a role through self-monitoring and immediate feedback mechanisms and may cultivate athletes’ stronger autonomy and intrinsic motivation by meeting the core psychological needs expounded by self-determination theory (Nuss et al., 2021). This, in turn, is expected to improve training compliance (both objective behavioral compliances, such as training frequency and duration, and subjective psychological input) and promote safe and efficient performance gains through the quantification and management of training load (Temm et al., 2022).

However, despite these plausible theoretical justifications, there is a significant gap between expectations and empirical evidence. There are three key limitations in the current mainstream research paradigm. First, existing studies have mainly focused on estimating the average treatment effect of digital activity trackers, largely ignoring the possibility of systematic heterogeneity of effect (Moe-Byrne et al., 2022). This “one-size-fits-all” approach to analysis obscures a key reality: the same “dose” of technology may produce different or even opposite results for adolescent athletes with varying initial fitness, skill, or psychological predispositions (Silva et al., 2021). For example, intense device use may motivate athletes at lower levels by enhancing perception, but for those approaching physiological limits, it may trigger overtraining and psychological exhaustion by impairing autonomy or causing stressful monitoring (Daley and Reardon, 2024). Second, the operationalized definition of “dose” relies heavily on retrospective self-reporting, which is susceptible to recall bias and social desirability, and fails to capture the fine-grained, objective quantitative information that device log data can provide (Patterson et al., 2021). Thirdly, one of the consequences is that the current research often regards the “technology-behavior-physiology” path as a black box, and fails to systematically examine the psychological factors such as training load (the key intermediary variable to transform behavior into physiological adaptation) and technology acceptance (based on TAM), which is an important moderator of technology adoption and continued use (Kao et al., 2019; Verstappen et al., 2021). This oversimplification severely limits our understanding of how mental processes interact with technology to influence outcomes, as well as who digital activity trackers are effective for (Neupert et al., 2022).

**Figure 1:**
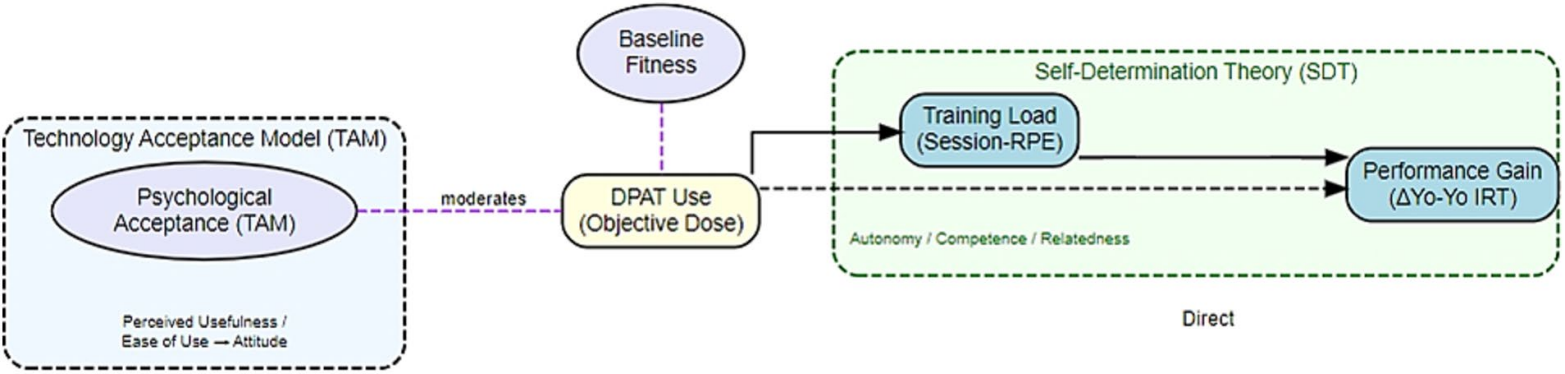
Integrated theoretical model incorporating the Technology Acceptance Model (TAM) and Self-Determination Theory (SDT). This conceptual model elucidates a hypothesized mechanism for the heterogeneous association between digital physical activity tracker (DPAT) use and performance improvement in adolescent athletes. The framework integrates TAM and SDT to describe the mental processes at different stages of technology engagement. Specifically, the TAM (shown in the blue dashed box) hypothesizes that the psychological acceptance formed by the athlete’s perception of usefulness and ease of use mediates the relationship between device availability and actual use behavior. The SDT (shown in the green dashed box) provides a motivational basis for the mediation pathway: the theory of consistent use of the device supports basic psychological needs (autonomy, competence, relevance), which in turn promotes training participation and thus performance. The model proposes that the association between DPAT usage and performance gain is both direct and indirect, mediated by the training load (session RPE). In addition, this dose–response relationship was moderated by baseline fitness levels and psychological acceptance, resulting in expected heterogeneity across performance quantiles.

In order to make up for these gaps, this study takes young football players as the object and carries out more elaborate exploration in methods. To address these gaps, we propose an integrated theoretical model (Figure 1) that combines the Technology Acceptance Model (TAM) and Self-Determination Theory (SDT). This integration serves a specific purpose: TAM helps predict which athletes are more likely to adopt and consistently use technology based on their perceived usefulness and ease of use. Subsequently, the SDT provides a framework for understanding how the continued use of such technologies can meet or hinder basic psychological needs (autonomy, competence, relevance), thereby explaining changes in long-term motivation, training behavior, and ultimately performance outcomes (Dwivedi et al., 2020; Flannery, 2017). This comprehensive model hypothesizes that the association of digital activity tracker dosage on performance is mediated by changes in training load, and that this association is mediated by the athlete’s psychological acceptance (from the TAM) and baseline characteristics such as initial physical fitness. Moreover, the intensity of these pathways may vary depending on the level of performance (Noorbhai et al., 2025). Based on this framework, this study aimed to answer four specific questions: (1) How does the objectively measured dose (frequency and duration) of digital activity tracker use differentially associated with the objective and subjective training compliance of youth football players? (2) Is the association between dose and performance improvement heterogeneous depending on the initial performance level of athletes? (3) Does training load mediate the association between dose and performance improvement? (4) Do athletes’ psychological acceptance of digital exercise trackers (TAM) and initial fitness level moderate the association between dose and adherence/performance, and can this moderating effect account for the observed heterogeneity?

To this end, we used a 12-week longitudinal cohort design with participants from multiple youth academies. We innovatively integrated continuous log behavioral data from the device application programming interface, high-intensity longitudinal subjective data collected through the empirical sampling method, and periodic standardized physiological test data (Poppe et al., 2025). In terms of analytical methods, this study goes beyond the limitations of the traditional linear mean model and uses quantile regression to systematically depict the differential associations of digital motion tracker dose on athletes in different locations of conditional performance distribution (such as low, medium and high quantiles) (Liu et al., 2024). We expect that this study will not only provide a precise empirical basis for the personalized application of digital training tools in youth sports but also promote the development of sports psychology literature by testing the integrated TAM SDT model. The research paradigm has shifted from seeking “average effect” to exploring “effect heterogeneity” and “individualized matching” (West et al., 2025).

## 2 Methods

### 2.1 Study design and participants

This study used a prospective longitudinal observational study design with three measurements: baseline (T0), week 6 (T1), and week 12 (T2). The subjects were recruited from U15 to U17 youth training echelons of four professional football clubs in East China. Using cluster sampling method, 156 male athletes were initially included after obtaining the informed consent of clubs and guardians. After excluding the athletes who could not participate in the whole training due to serious injuries, the final analysis sample included 142 athletes (age: average 16.2 years, standard deviation 1.1 years; training years: average 5.3 years, standard deviation 2.4 years), distributed in 12 training teams. Ex ante power analysis by Monte Carlo confirmed that the statistical power of the current sample to detect a significant effect at the major quantile (*τ* = 0.25, 0.50, 0.75) was more than 80% for moderate effect sizes (Shieh, 2018).

### 2.2 Variable measurement and operationalization

#### 2.2.1 Digital tracker use dose (independent variable)

*Device*: The Garmin Forerunner 255 watch is uniformly available.

##### 2.2.1.1 Objective Dose (API-derived)

*Use Frequency*: Number of days per week that the device was worn and recorded for at least one effective session (more than 30 min).

*Use Duration*: the total number of minutes of effective training per week recorded by the equipment.

*Cumulative Dose*: The total days of use and the total duration of use from the baseline period were calculated at T1 and T2, respectively.

#### 2.2.2 Training adherence and performance (dependent variables)

*Objective Training Adherence*: derived from equipment data and calculated as the training session completion rate (sessions completed/scheduled) and the average training session duration per two-week period.

*Subjective Training Adherence*: measured by experience sampling. Athletes reported perceived exertion scores (RPE, Borg CR-10 scale) and concentration (3 items, 7-point Likert scale, Cronbach’s coefficient *α* = 0.88) within 30 min after the end of training. Biweekly averages were calculated (Khanshan et al., 2021).

##### 2.2.2.1 Athletic Performance

Performance of Yo-Yo intermittent recovery test: test at T0, T1 and T2. Record the total distance completed. Performance improvement was calculated as ΔYo-Yo (T2 test score minus T0 test score) (Póvoas et al., 2016).

*Composite Performance Index (CPI)*: To enhance validity, the 30-meter dash, the Illinois agility test, and the reverse vertical jump height were measured simultaneously. The test scores of T0 and T2 were standardized and subjected to principal component analysis. The first principal component (accounting for 65% of the variance) was extracted as the composite performance index and its pretest-posttest difference (ΔCPI) was calculated (Wagner et al., 2019).

#### 2.2.3 Mediator and moderator variables

*Training Load (Mediator)*: a calculation method based on the score of conscious exertion in a single training session was used. Weekly training load (arbitrary unit) = *Σ* (training session duration [min] × training session perceived exertion score). The average weekly load was calculated for T0 to T1 and T1 to T2, respectively (Gaudino et al., 2015). We acknowledge that the use of the RPE as the only measure captures internal perceptual loading but does not distinguish between physiological and psychological stress components or account for mechanical or neuromuscular loading.

*Psychological Acceptance (Moderator)*: At T0, it was measured by the adapted version of the Technology Acceptance Model questionnaire, which included three subscales: perceived usefulness (4 items, *α* = 0.91), perceived ease of use (4 items, *α* = 0.89) and attitude towards use (3 items, α = 0.87). The total score was taken as the mean score of each subscale (Ruiz-Figueroa et al., 2024). The scale uses a validated TAM instrument. Confirmatory factor analysis indicated a good fit of the model (CFI = 0.96, RMSEA = 0.06), supporting its construct validity.

#### 2.2.4 Covariates

Age, years of training, baseline Yo-Yo interval recovery test scores (for quantile stratification), injury history (number of weeks lost to injury in the past 6 months), and club/team dummy variables.

### 2.3 Statistical analysis

A multimethod sequential statistical analysis strategy was used in this study. Descriptive statistical analysis was first performed to describe the distribution of sample characteristics and key variables (Table 1). Core analysis employed quantile regression to model the association between cumulative duration of digital motion tracker use and performance improvement (ΔYo-Yo interval recovery trials) at quantiles of *τ* = 0.10, 0.25, 0.50, 0.75, 0.90. All models controlled for baseline performance, age, years of training, injury history, and club affiliation (Table 2) (Lee et al., 2021). Robust standard errors were obtained using 500 repeated bootstraps. On this basis, the quantile regression framework is extended to test its mechanism: quantile mediation model is used to evaluate the mediation effect of training load. The method estimates indirect effects (ab) at specific quantiles of the outcome distribution by bootstrapping, which does not assume a normal distribution of indirect effects. The moderating effect of psychological acceptance was tested by introducing an interaction term (dose × moderator) in the quantile regression model, and the significance of the interaction was assessed by bootstrap confidence intervals (Table 3) (Li et al., 2022).

**Table 1:** Sample characteristics, device use dose, and descriptive statistics of key variables (*N* = 142).

Variable category	Specific variable	Mean (M)/percentage (%)	Standard deviation (SD)	Range/IQR
Demographics	Age (years)	16.21	1.07	14.8–17.9
	Training experience (years)	5.34	2.42	2–11
Baseline performance	Yo-Yo IRT (meters)	1864.5	432.6	920–2,840
	Composite performance index (*Z*-score)	0.01	1.00	(−2.35, 2.18)
Dose (12-week cumulative)	Total usage days	67.8	14.2	28–84
	Total usage duration (hours)	145.6	41.3	48–256
Outcome variables	ΔYo-Yo IRT (meters)	280.4	178.9	−120-720
	Δ Composite performance index	0.52	0.78	(−1.10, 2.65)
	Objective adherence (completion rate %)	88.5%	9.8%	52–100%
	Subjective effort (RPE)	7.2	1.5	4–10
Mechanism variables	Avg. weekly training load (AU)	3,850	1,120	1,560–6,540
	Psychological acceptance total score	4.15	0.76	(2.1, 5.8)

IQR, Interquartile Range; AU, Arbitrary Units.

**Table 2:** Quantile regression estimates for the effect of cumulative use duration on performance gain (ΔYo-Yo IRT).

Predictor	*τ* = 0.10	*τ* = 0.25	*τ* = 0.50 (median)	*τ* = 0.75	*τ* = 0.90
Cumulative duration (hours)	1.85 (0.62)**	2.41 (0.48)***	1.22 (0.40)**	0.68 (0.37)†	−0.21 (0.51)
Baseline Yo-Yo (100 m)	−17.8 (5.1)***	−22.1 (4.0)***	−25.3 (3.2)***	−26.8 (3.0)***	−30.4 (4.1)***
Age (years)	15.2 (18.1)	12.8 (14.0)	10.5 (11.2)	8.3 (10.5)	5.1 (14.8)
Training experience (years)	8.9 (6.2)	10.1 (4.8)*	11.5 (4.0)**	13.0 (3.8)***	14.8 (5.2)**
Injury history (weeks)	−25.6 (11.3)*	−20.1 (8.8)*	−15.3 (7.2)*	−10.8 (6.8)	−5.2 (9.5)
Club dummies	Included	Included	Included	Included	Included
Pseudo *R*^2^	0.28	0.32	0.35	0.33	0.29

*N* = 142. Table presents unstandardized regression coefficients with bootstrapped standard errors (500 replications) in parentheses. The model estimates the effect on ΔYo-Yo IRT (meters) at the specified quantiles (*τ*). ***p < 0.001, **p < 0.01, *p < 0.05, †*p* < 0.10.

**Table 3:** Quantile-specific mediation and moderation analyses.

Analysis & path	Quantile (*τ*)	Effect estimate	95% Boot CI	Test statistic/index
A. Mediation via training load				
Indirect effect (ab)	*τ* = 0.25	0.89	[0.32, 1.65]	–
	*τ* = 0.50	0.51	[0.15, 1.02]	–
Proportion mediated	*τ* = 0.25	36.9%	–	–
B. Moderation by acceptance				
Interaction coef. (dose × accept)	*τ* = 0.25	0.95	[0.18, 1.77]	*t* = 2.42
	*τ* = 0.50	0.41	[−0.23, 1.09]	*t* = 1.25
C. Moderation by baseline fitness				
Interaction coef. (dose × baseline)	*τ* = 0.25	−0.85	[−1.62, −0.15]	*t* = −2.39
	*τ* = 0.50	−0.51	[−0.98, −0.05]	*t* = −2.18

*N* = 142. CI = Bias-corrected bootstrap confidence interval (5,000 reps). Proportion mediated = indirect effect/total effect.

To ensure the robustness of the results, a number of tests were included in the analysis: using frequency of use as an alternative dose indicator to re-estimate the master model; re-analyzing after eliminating extreme dose users; and using a panel fixed-effect quantile regression model to control for unobservable, non-time-varying heterogeneity (Table 4) (Dai et al., 2020). To assist in the substantive interpretation of the quantile regression results, a complementary analysis was performed by dividing the sample into two subgroups of “low baseline performance” and “high baseline performance” according to the median of the baseline Yo-Yo intermittent recovery test performance and testing their dose–response patterns separately. Data visualization is essential for interpreting complex results, and the primary plots include the distribution of the primary variables, the trajectory of the dose coefficient over the full quantile range to illustrate the heterogeneity of the effect, and the dose–response plot at selected quantiles to depict the potential nonlinear relationship. To further validate the interpretation that the low quantiles of performance improvement correspond to those with lower baseline performance, a supplemental analysis was also conducted by grouping by median baseline performance. All statistical analyses were completed using Stata 18.0 and R 4.3.0 software, and significance tests were performed using two-sided tests with the alpha level set at 0.05.

**Table 4:** Robustness checks of the dose–response relationship across quantiles (ΔYo-Yo IRT).

Specification/model	Sample/key variation	*τ* = 0.25	*τ* = 0.50	*τ* = 0.75
1. Main model	Cumulative duration (hrs)	2.41 (0.48)*	1.22 (0.40)	0.68 (0.37)†
2. Alt. dose metric	Average weekly duration	3.12 (1.02)	1.85 (0.85)*	0.91 (0.78)
3. Sample restriction	Excl. extreme users (*n* = 134)	2.05 (0.51)*	1.10 (0.43)*	0.61 (0.40)
4. Additional control	+Baseline motivation	2.35 (0.50)*	1.19 (0.42)	0.66 (0.39)†
5. Panel FE QR	Within-individual estimator	2.18 (0.71)	1.35 (0.59)*	0.72 (0.55)

All models control for baseline performance, age, training experience, injury history, and club. Bootstrap SEs in parentheses.

## 3 Results

### 3.1 Descriptive statistics and preliminary analyses

The final analysis sample included 142 male youth football players (age: mean 16.2 years, SD 1.1 years; training years: mean 5.3 years, SD 2.4 years). Detailed descriptive statistics for all study variables are presented in Table 1. During the 12-week intervention period, participants used the digital motion tracker for an average of 67.8 days (14.2 standard deviations), with a cumulative duration of approximately 145.6 h (41.3 standard deviations). There was a significant improvement in athletic performance, with a mean score of 280.4 meters (standard deviation 178.9) on the ΔYo-Yo Interval Recovery Test. Objective training compliance was high (mean 88.5% completion rate), and subjective reports indicated moderate to high effort (mean 7.2 perceived exertion score). The distribution of key variables, including performance improvement and equipment dose, is shown in Figure 1.

**Figure 2:**
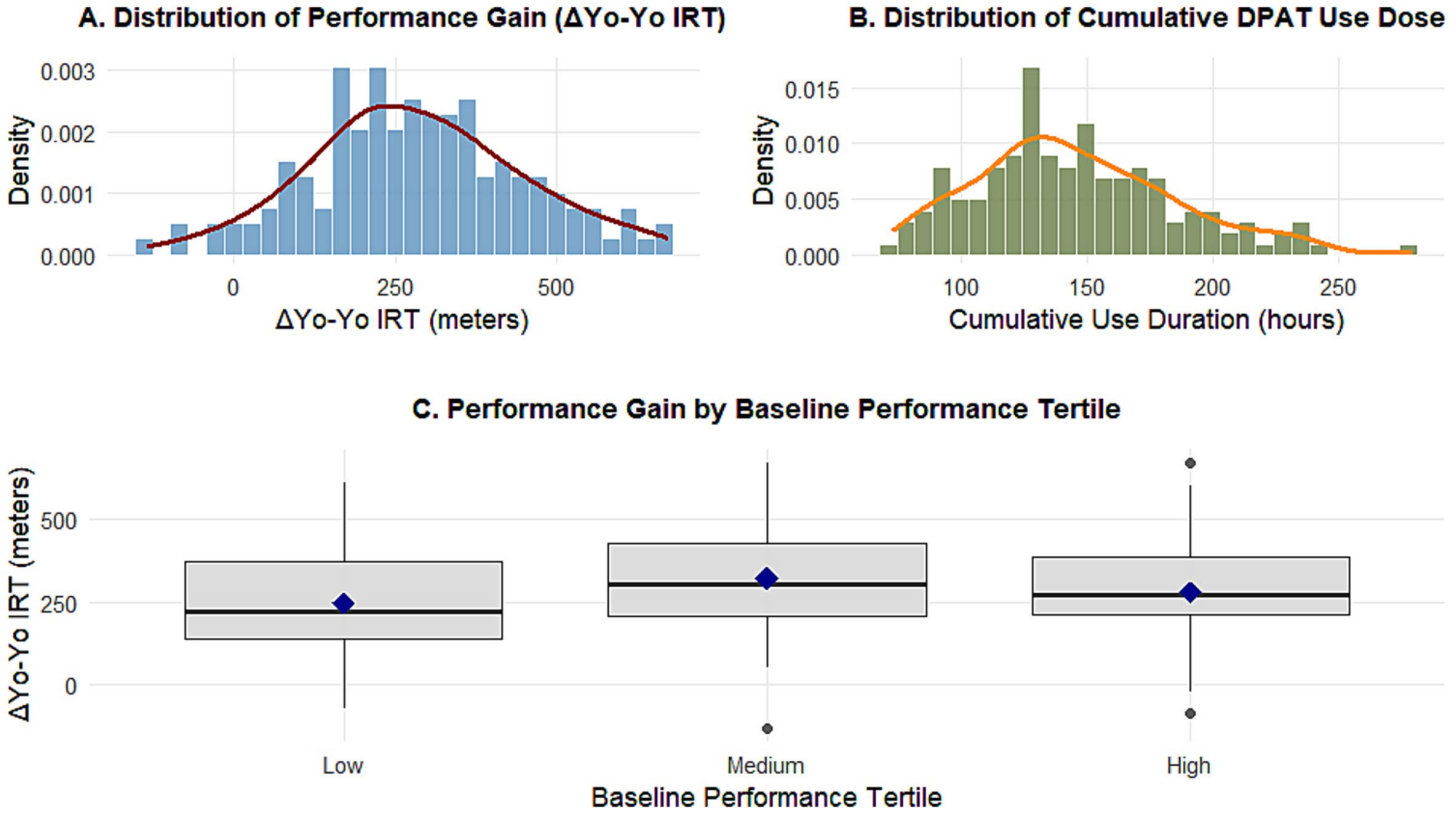
Distributions of key variables and preliminary heterogeneity. **(A)** The density distribution of performance improvement (ΔYo-Yo intermittent recovery test) is approximately normal and slightly right-skewed. **(B)** The density distribution of the cumulative duration of digital motion tracker use is highly variable among individuals. **(C)** The boxplot shows that athletes in the lowest tertile group of baseline performance achieved the greatest median and mean improvement, indicating diminishing returns for better performers.

Figure 2 shows the distribution of key variables. Figure 2A shows that the distribution of ΔYo-Yo intermittent recovery test performance improvement is approximately normal, with a slight right deviation; Figure 2B shows that there is a large variability in cumulative dose among different athletes. Notably, when participants were divided into three groups based on baseline performance, the group with the lowest baseline performance had the greatest improvement in average performance (see Figure 2C), providing preliminary evidence of effect heterogeneity. This was confirmed by a supplemental analysis performed by grouping by baseline performance (low versus high performance), which showed a steeper slope of the dose-performance curve in the low baseline performance group.

### 3.2 Heterogeneous effects of dose on performance gain

Quantile regression analysis revealed significant and statistically significant heterogeneity in the association between cumulative duration of digital motor tracker use and performance improvement (ΔYo-Yo Interval Recovery Test). As shown in Table 2, there is a significant difference in the distribution of conditions for performance improvement. This association was strongest at the low quantile (*τ* = 0.25: *B* = 2.41, bootstrap standard error = 0.48, *p* < 0.001), indicating that for athletes with relatively small gains, each additional hour of equipment use was associated with an approximate 2.4-meter gain in their Yo-Yo Interval Recovery test performance. The association was attenuated at the median quantile (*τ* = 0.50: *B* = 1.22, bootstrap standard error = 0.40, *p* < 0.01) and only marginally significant at the 75th percentile (*τ* = 0.75: *B* = 0.68, bootstrap standard error = 0.37, *p* = 0.07). Crucially, at the 90th percentile, representing the athletes with the greatest improvement, the association was minimal and not significant (*B* = 0.21, self-service standard error = 0.51, *p* = 0.68).

**Figure 3:**
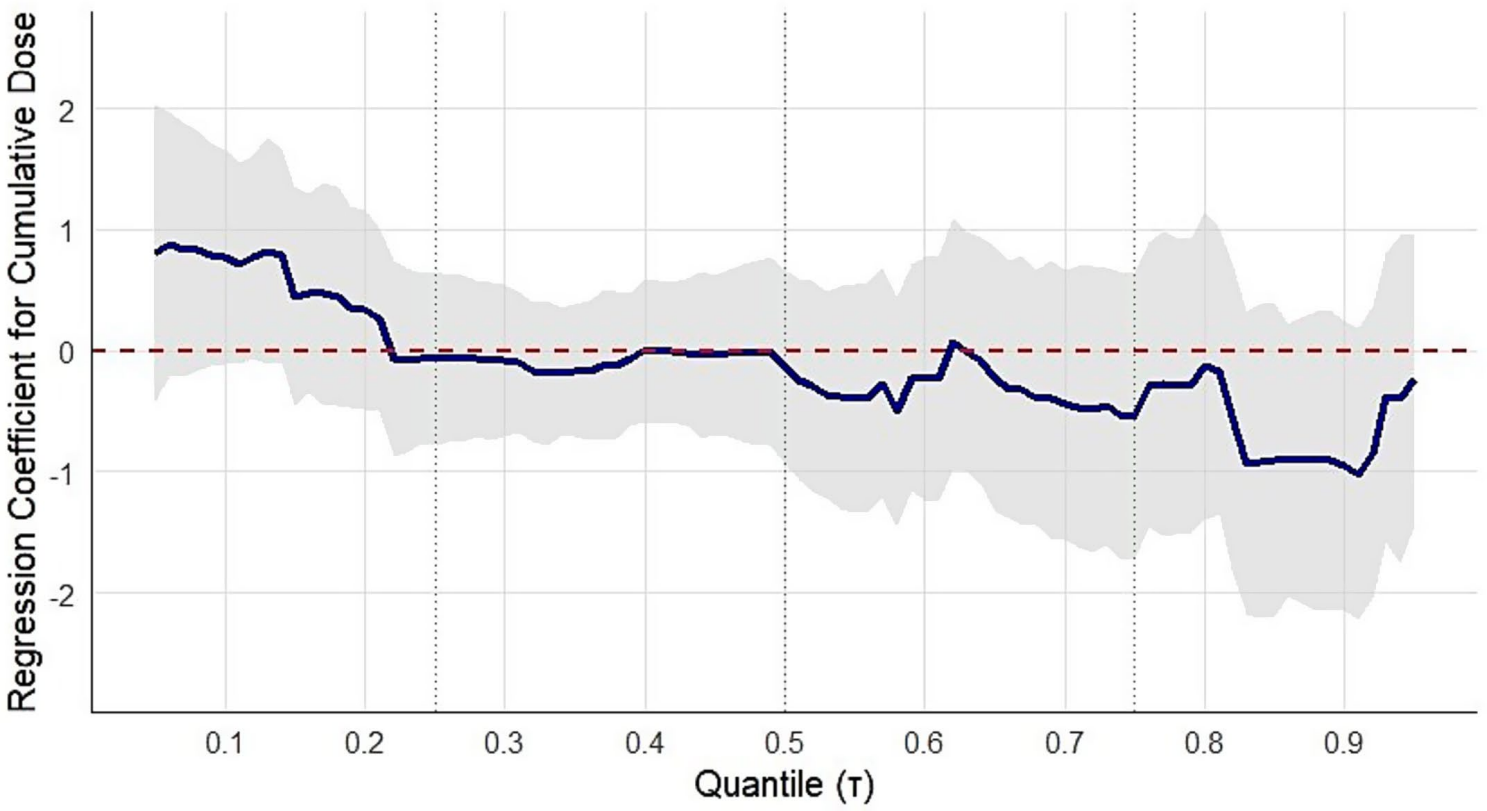
Trajectory of dose-effect coefficients across quantiles. The blue solid line depicts the quantile regression coefficients (*τ* = 0.05 to 0.95) of the cumulative digital motion tracker using the pair ΔYo-Yo intermittent recovery test; the gray band is the 95% bootstrap confidence interval. The red dashed line marks the zero value. Coefficients decrease monotonically from significant positive values at the low quantile to insignificant values at the high quantile, indicating that the positive association is strongest for athletes with the least lift.

Figure 3 visually demonstrates this heterogeneity by plotting the trajectory of the dose coefficient change for a full quantile range (*τ* from 0.05 to 0.95). The coefficients show a clear monotonically decreasing trend, and the point estimates and their 95% confidence intervals are well above zero until *τ* ≈ 0.80, after which they contain zero. This pattern strongly confirms that the performance-enhancing associations of digital activity tracker use are most significant for athletes at the lower end of the performance-enhancing distribution and are attenuated for athletes who achieve greater improvements. In a substantive sense, this suggests that athletes with smaller final improvement (generally those with lower baseline levels, as supported by baseline Yo-Yo scores with negative coefficients at all quantiles) had greater equipment usage, which was most strongly associated with better outcomes. Baseline performance was a consistent negative predictor at all quantiles, confirming the statistical phenomenon of diminishing returns for athletes with higher initial levels.

**Figure 4:**
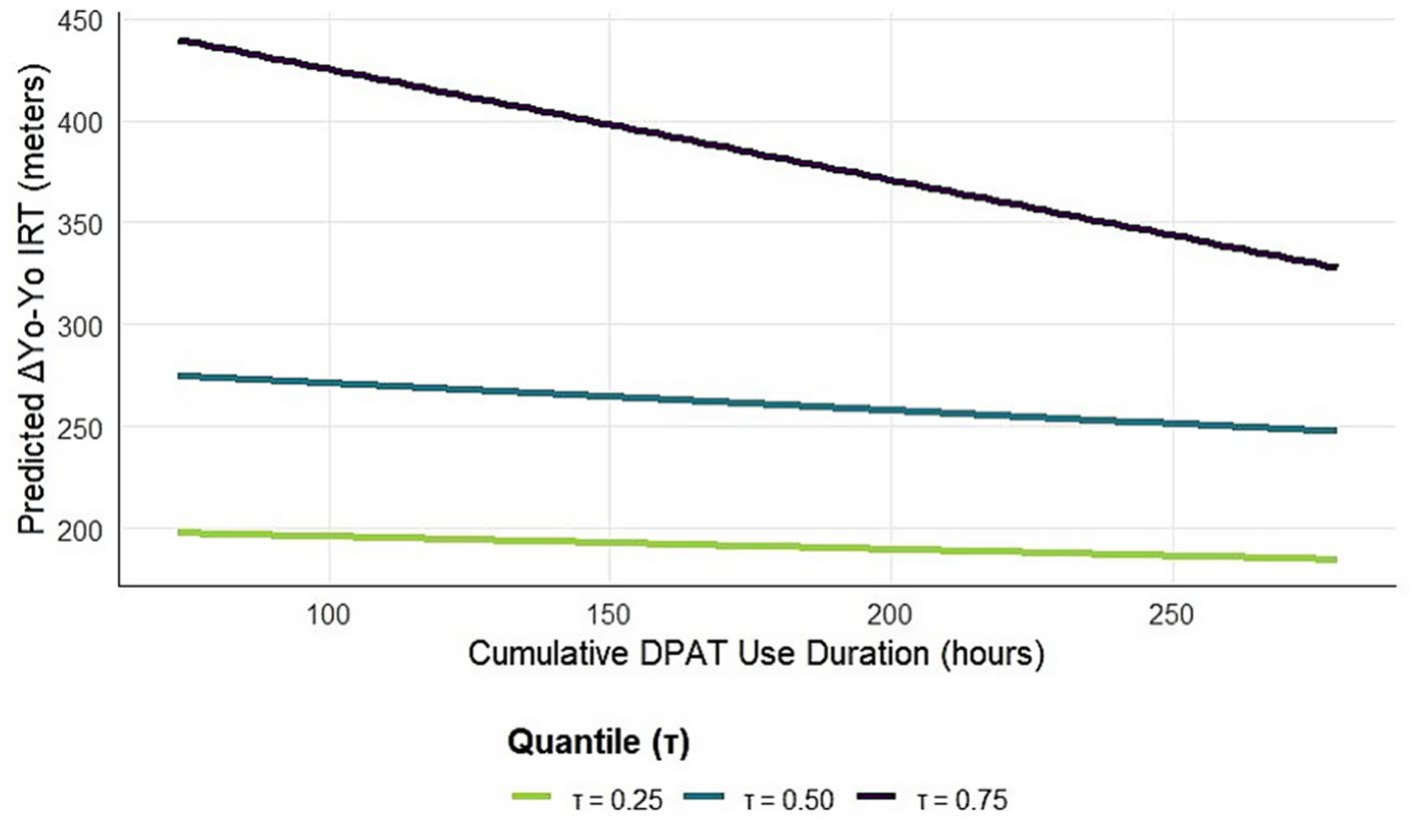
Predicted dose–response curves at selected quantiles. The lines in the figure show the predicted ΔYo-Yo intermittent recovery test values for the cumulative duration of use at the *τ* = 0.25, 0.50, and 0.75 quantiles, with all covariates fixed at the median. *τ* = 0.25 (low risers) has the steepest slope and flattens out as the quantile rises, confirming the diminishing returns and nonlinear nature of the dose–response relationship.

Figure 4 further illustrates this relationship by presenting predicted dose–response curves at three representative quantiles (*τ* = 0.25, 0.50, 0.75), with all other covariates fixed at the median. The curves show an overall positive but non-linear relationship, with the steepest slope at the low quantile (*τ* = 0.25) and the gentlest slope at the high quantile (*τ* = 0.75). The phenomenon of diminishing marginal returns is clearly visible in the figure, especially after more than about 150 h of cumulative use.

### 3.3 Robustness of the heterogeneous dose–response relationship

The pattern of identified heterogeneous effects remained robust across a range of methodological changes and sample limitations (Table 4). When the dose was manipulated to the weekly average duration instead of the cumulative duration, the decay pattern of the coefficients across the quantile remained the same, despite the increased standard error due to data aggregation. Excluding the extreme dose users (highest and lowest 5%), the coefficients weakened slightly but did not change the direction of the quantile trend or the pattern of statistical significance. After controlling for potential psychological confounding variables baseline motivation, the coefficients obtained were nearly identical to the master model. Most notably, a panel fixed-effects quantile regression model that considered all individual heterogeneity that did not change over time produced qualitatively consistent and statistically significant results at the median quantile (*B* = 1.35, *p* < 0.05), which strengthened the evidence in favor of intra-individual association. Although concerns about reverse causation or time-varying confounders have not been eliminated.

### 3.4 Mechanisms underlying heterogeneous effects: mediation and moderation

Mechanism analysis provided insights into the potential sources of observed heterogeneity in association (Table 3). First, training load played a significant mediating role, but its effect was quantile specific. The indirect effect of dose on the performance of ΔYo-Yo intermittent recovery test by increasing training load was significant at *τ* = 0.25 (ab = 0.89, 95% self-service confidence interval [0.32, 1.65]) and *τ* = 0.50 (ab = 0.51, 95% self-service confidence interval [0.15, 1.02]). They account for about 37 and 42% of the total effect, respectively. This suggests that a significant portion of the association of digital activity trackers are observed by facilitating greater (and potentially better managed) training loads for athletes with moderate to low lift. No significant mediation effect was found at *τ* = 0.75.

Psychological acceptance significantly moderates the dose–response relationship, but only at the low quantile (*τ* = 0.25). The positive and significant interaction term (*B* = 0.95, 95% self-help confidence interval [0.18, 1.77], *p* < 0.05) indicated that among athletes with lower improvement, the performance improvement associations of equipment use were more significant for those with higher initial psychological acceptance.

Most critically, the initial fitness level itself is a powerful moderator that helps explain the major heterogeneity. A significant negative interaction between dose and baseline Yo-Yo intermittent recovery test performance was observed at *τ* = 0.25 (*B* = 0.85, *p* < 0.05) and *τ* = 0.50 (*B* = 0.51, *p* < 0.05). This suggests that the positive association of device use was significantly attenuated for athletes with higher fitness levels at the start of the study. This finding directly explains the observed pattern of coefficient decay across quantiles: athletes in the low quantile of performance improvement were more likely to have lower baseline fitness levels, and for this group, digital sport tracker use showed the strongest positive association. Conversely, athletes who performed better (at the high percentile) generally had higher initial fitness levels, so the association was weakened.

## 4 Discussion

This study is the first to systematically examine the heterogeneous association between digital sport trackers and training compliance and sport performance of youth football players by integrating objective device data with high-density longitudinal subjective reports and using quantile regression. The core findings reveal a clear pattern: the association effect of digital activity trackers is not homogeneous, but there are significant and systematic individual differences, and the strength and direction of this association are highly dependent on the initial performance level of athletes. Specifically, for athletes with low baseline performance and small performance improvement, equipment use showed a significant positive association, while for athletes with high baseline performance and large performance improvement, the association was greatly weakened or even disappeared. The heterogeneity of the dose–response relationship was partly explained by the mediating effect of training load and the moderating effect of baseline physical fitness and psychological acceptance. The following sections discuss these findings in depth, illustrating their theoretical significance, practical value, and limitations of this study.

### 4.1 Interpretation of main findings and theoretical dialogue

The most important finding of this study is the existence of a heterogeneous pattern of effects of digital motion trackers depending on the initial level of performance. This finding is in critical dialogue with and extends theories of sport psychology. It challenges the “one-size-fits-all” assumption prevalent in the digital health intervention literature, and such studies often seek and report average treatment effects (Vitger et al., 2021). Our results contrast with those of previous studies that reported consistent positive effects of wearables on motivation or performance in youth athletes, possibly because those studies relied on mean-based models (e.g., analysis of variance, standard linear regression), masking underlying distributional heterogeneity (Delves et al., 2021). Our quantile regression method reveals a reality that is consistent with the principle of diminishing marginal returns and interindividual response variability in exercise science (Hecksteden et al., 2017). More importantly, it provides a nuanced perspective on self-determination theory. For low-performing athletes, digital activity trackers may provide structured self-monitoring and immediate feedback that they previously lacked, significantly enhance the scientificity of their training, and may meet their needs for competence through clear progress visualization and autonomy through self-regulation training. Thereby generating substantial marginal returns (Ridgers et al., 2016). On the contrary, for high-level athletes, their training may be highly structured and close to the physiological limit, and additional technical feedback may lead to information overload, duplication with coach feedback, and even lead to “analysis paralysis” and psychological pressure caused by over-examination of data, which may weaken their sense of autonomy or create external control. Thus, counteracting the positive effect (Brenner et al., 2024).

This nuanced perspective expands the application of previous self-determination theory in the field of sports technology. Previous studies usually suggest that there is a general positive link between autonomy and motivation supported by technology. Failure to consider this association may be attenuated or even reversed for athletes who are already highly autonomous and competent (Davis et al., 2022). In addition, the moderating effect of the technology acceptance model was significant only at the low quantile. This underscores that for the subpopulations most likely to benefit from the intervention (the smaller lifters, often those with a lower baseline), individual perceptions of usefulness and ease of use are key psychological filters for translating their technology exposure into actual gains (Shin et al., 2019). For high performance athletes who may already be close to optimal training, the effect of technical attitude appears to be more limited, suggesting that motivational strength or other factors may dominate at this level (Powell et al., 2022).

### 4.2 The dose–response relationship and underlying mechanisms

This study identified a non-linear dose–response relationship whose shape varies with the conditional distribution (Gralla et al., 2019). The mediating effect of training load was significant only at the low and middle quantiles, revealing a key behavioral pathway: for low and middle level athletes, the device may work primarily by promoting more active and regular training participation (i.e., higher objective dose), which translates into a controlled increase in training load. Ultimately, it will promote performance improvement (Debien et al., 2022). This suggests that, for this group, part of the value of digital motion trackers lies in overcoming participation barriers and optimizing training inputs (Vajravelu and Arslanian, 2021).

This finding partially supports, but also revises, previous research that viewed training load as a common mediator between wearable device use and performance (Clemente et al., 2021). Our quantile-specific mediation results suggest that this mechanism is not universal but conditionally relevant (Hayes and Rockwood, 2017). The lack of significant mediating effects at the high quantiles suggests that for elite adolescent athletes, performance improvement may be less dependent on device-facilitated increases in quantifiable training loads and more dependent on qualitative aspects such as technical and tactical refinement, psychological conditioning, or recovery optimization. These are factors that are not fully captured by the perceived exertion score in a single training session (Symons et al., 2023). Indeed, we acknowledge a key measurement limitation: using the RPE as the sole proxy for training load captures an athlete’s internal perception of effort, but does not distinguish between physiological and psychological stress, nor does it allow for objective external loads (e.g., distance, acceleration). This may be the reason why the mediation pathway is not significant for high-level athletes, and the progress of high-level athletes may depend more on factors such as training quality or tactical refinement. Therefore, our results call for a functional evolution of digital activity tracker design from a primary focus on “promoting behavioral compliance” to a function that can “optimize training quality” and “promote recovery regulation” to meet the needs of high-performance athletes. This is a shift that has been hinted at but not fully explored in the current sports technology literature (Kaufman et al., 2025).

### 4.3 Theoretical contributions

This study has made multiple theoretical contributions to the fields of sport psychology and sport technology. Firstly, in terms of methodology, we systematically introduced quantile regression into the evaluation of sports technology effect, which promoted the research paradigm in this field to shift from focusing on “average treatment effect” to describing “effect heterogeneity” (O’Keeffe et al., 2020). This encourages future research to look beyond “does it work?” To delve into the question, “For whom, under what conditions, and to what extent?” (Jedwab et al., 2021). Second, by integrating TAM and SDT, we constructed and partially validated a multi-level mental framework. The TAM identifies the initial acceptance of the individual as the key filter for technical input, and the SDT explains the motivational processes through which continued use may influence training behavior and outcomes. The integration model provides a novel and testable framework for future research on athlete technology interaction (Gasser et al., 2025). Thirdly, the results show that the association effect of digital intervention is not determined unilaterally by technology itself, but the product of the interaction between user characteristics (initial level, psychological acceptance) and technical characteristics, which provides empirical support for the application of “person-technology matching” theory in sports situations (Zhou and Daud, 2024).

### 4.4 Practical implications

These findings have direct implications for youth footballtraining practices. For coaches and trainers, uniform expectations of the benefits of digital activity trackers should be abandoned in favor of differentiated promotion and management strategies (Urban et al., 2025). For younger athletes or athletes at the basic stage, they can be actively encouraged to use data for personal goal setting and motivation (Freire et al., 2025). The key is that for this group, efforts to improve psychological acceptance (such as demonstrating usefulness and ensuring ease of use) may be particularly important for releasing benefits (Drehlich et al., 2020). For elite young athletes, the auxiliary and instrumental nature of equipment should be emphasized, and it should be integrated into the existing training system to avoid data-dominated decision-making, while closely monitoring the psychological response to prevent technical stress (Jordalen et al., 2016). For sports technology developers, efforts should be made to create more adaptable algorithms and feedback interfaces, such as more encouraging and educational basic feedback for novice users and more streamlined advanced analysis matched with high-level training goals for elite users (Swain et al., 2024). For Academy policy makers, when procuring and deploying such technologies, it is important to recognize that the cost-effectiveness of the benefits may vary across subgroups. Investment should be matched with educational support to maximize technological acceptance and effectiveness.

### 4.5 Limitations and future directions

This study has several limitations that should be taken into account when interpreting the findings. First, and most importantly, despite controlling key covariates and employing a fixed-effects model, the observational longitudinal design was unable to determine causality. Dose of device use was self-selected, and while we controlled baseline motivation, other unmeasured confounding variables (e.g., inherent self-regulation, social support) may affect both device use and performance gains. Therefore, our findings should be interpreted as associations, not causal effects. Future randomized controlled trials with stratified doses are needed to confirm the causal effect and determine the optimal dose range for different subgroups of athletes. Second, the use of RPE as the only measure of training load is a limitation, as it only captures the perceptual aspects of the load. Future studies should combine objective external load measures (e.g., GPS or accelerometer) and physiological measures (e.g., heart rate variability) to gain a more complete understanding of how training load mediates the relationship between technology use and performance. Third, we focused on behavioral compliance and physical performance. Future studies should include richer psychometric measures (e.g., basic psychological need satisfaction, motivation regulation, burnout in self-determination theory) and psychophysiological measures (e.g., heart rate variability as a marker of stress/recovery) to more fully reveal the psychological and biopsychosocial mechanisms underlying the observed effects. Finally, while dose was defined objectively based on duration, we did not distinguish between the specific functions used (e.g., viewing data, setting goals, social sharing), which may have different effects.

Based on this, future studies can: 1) carry out randomized controlled trials with dose stratification to test causal effects and optimal dose ranges more rigorously; 2) use mixed methods combined with qualitative interviews to gain insight into the experience and perception of athletes at different levels of using digital motion trackers; 3) implementing a longer follow-up to examine the sustainability of the effects and the potential decay of “novelty effects”; 4) using path analysis or latent variable growth models to explicitly test the integrated technology acceptance model and self-determination theory model in a larger sample; 5) Explore whether AI-driven personalized feedback can mitigate or even eliminate the heterogeneity observed in this paper, thus enabling true personalized training support.

## 5 Conclusion

This study reveals that the association between digital motion trackers and youth football player performance presents a complex, heterogeneous picture. The observed associations were not universal but were significantly biased toward better outcomes for athletes with lower initial performance and greater room for improvement, due in part to increased training loads. Athlete psychological acceptance and initial fitness were key moderators in shaping this relationship, supporting a complete TAM-SDT framework to account for this heterogeneity. While this study provides strong evidence of heterogeneous associations and their possible mechanisms, the nonrandomized design precludes causal inference. These findings suggest the need for a critical and precise perspective when embracing sports technology. The value of technology lies not in its inherent complexity, but in its proper matching with the psychological characteristics and performance level of individual athletes. Future research and practice of digital interventions in sports science should shift from seeking universal “technological solutions” to exploring evidence-based and personalized “technological adaptation strategies”.

## Data Availability

The raw data supporting the conclusions of this article will be made available by the authors, without undue reservation.
